# Far-Infrared Therapy Accelerates Diabetic Wound Healing via Recruitment of Tissue Angiogenesis in a Full-Thickness Wound Healing Model in Rats

**DOI:** 10.3390/biomedicines9121922

**Published:** 2021-12-15

**Authors:** Rong-Fu Chen, Keng-Fan Liu, Su-Shin Lee, Shu-Hung Huang, Yi-Chia Wu, Yun-Nan Lin, Chun-Ting Wang, Yur-Ren Kuo

**Affiliations:** 1 Department of Surgery, Division of Plastic Surgery, Kaohsiung Medical University Hospital, Kaohsiung 80756, Taiwan; 1060008@kmuh.org.tw (R.-F.C.); cell77821@gmail.com (K.-F.L.); 830170@kmuh.org.tw (S.-S.L.); 900091@kmuh.org.tw (S.-H.H.); 970452@kmuh.org.tw (Y.-C.W.); 980277@kmuh.org.tw (Y.-N.L.); chuntingb20120@gmail.com (C.-T.W.); 2 Regenerative Medicine and Cell Therapy Research Center, Department of Biotechnology, Faculty of Medicine, College of Medicine, College of Life Science, Kaohsiung Medical University, Kaohsiung 80756, Taiwan; 3 Department of Biological Sciences, National Sun Yat-Sen University, Kaohsiung 80424, Taiwan; 4 Orthopaedic Research Center, Faculty of Medicine, College of Medicine, Kaohsiung Medical University, Kaohsiung 80756, Taiwan; 5 Academic Clinical Programme for Musculoskeletal Sciences, Duke-NUS Graduate Medical School, Singapore 169857, Singapore

**Keywords:** far infrared, diabetic wound healing, angiogenesis

## Abstract

Far-infrared ray (FIR) therapy has been applied in the tissue regeneration field. Studies have revealed that FIR could enhance wound healing. However, the biological effects of FIR on diabetic wounds remain unclear. Our study aims to investigate whether FIR could accelerate diabetic wound healing and analyze the biomechanisms. A dorsal skin defect (area, 6 × 5 cm^2^) in a streptozotocin (STZ)-induced diabetes rodent model was designed. Thirty-two male Wistar rats were divided into 4 groups (*n* = 8 each subgroup). Group 1 consisted of sham, non-diabetic control; group 2, diabetic control without treatment; group 3, diabetic rats received 20 min FIR (FIR-20, 20 min per session, triplicate/weekly for 4 weeks) and group 4, diabetic rats received 40 min FIR (FIR-40, 40 min per session, triplicate in one week for 4 weeks). The wound healing was assessed clinically. Skin blood flow was measured by laser Doppler. The vascular endothelial growth factor (VEGF), 8-hydroxy-2-deoxyguanosine (8-OHdG), eNOS, and Ki-67, were analyzed with immunohistochemical (IHC) staining. Laser Doppler flowmetry analysis of the blood flow of wounding area revealed the blood flow was higher in diabetic rats who received 40 min FIR (FIR-40) as compared to that in FIR-20 group. The wounding area was significantly reduced in the FIR-40 group than in the diabetic control groups. Histological findings of peri-wounding tissue revealed a significant increase in the neo-vessels in the FIR-treated groups as compared to the controls. IHC staining of periwounding biopsy tissue showed significant increases in angiogenesis expressions (VEGF, eNOS, and EGF), cell proliferation (Ki-67), and suppressed inflammatory response and oxygen radicles (CD45, 8-OHdG) expressions in the FIR-treated groups as compared to that in controls. Treatment with the optimal dosage of FIR significantly facilitated diabetic wound healing and associated with suppressed pro-inflammatory response and increased neovascularization and tissue regeneration.

## 1. Introduction

Chronic wounds occur commonly and reduce the quality of life of those affected, posing a relevant clinical and socioeconomic burden. Skin wound healing is a complex multistep process involving the coordination of activities of multiple tissue and cell types [1]. Diabetic foot ulcers are a significant intricacy of diabetes mellitus and are probably the major component of the diabetic foot [2,3]. Re-epithelialization, a vital cycle in the beginning phase of wound healing, is the consequence of the movement and multiplication of keratinocytes in the epidermal layer of the skin around the injury. Multiple main signaling transduction is enacted during the process of wound healing; these pathways incorporate the mitogen-activated protein kinase-related pathway, Wnt/β-catenin, and vascular endothelial growth factor (VEGF) pathways [1,4,5]. 

Photobiomodulation (PBM) therapy is a form of light therapy that uses non-ionizing forms of light sources in the visible and infrared spectrum, including lasers, LEDs, and broadband light [6,7]. PBM can bring beneficial therapeutic effects, including relief of pain or inflammation, immunomodulation, and promotion of tissue regeneration and wound healing [1,8]. Recently, much attention has been paid to that far-infrared radiation (FIR) may promote wound healing by stimulating the secretion of transforming growth factor β1 (TGF-β1) or activating fibroblasts [9]. However, FIR still knows little about the details of the molecular mechanisms of wound healing.

Infrared radiation transfers energy to surrounding tissues and is perceived as heat by thermoreceptors in the adjacent skin [10]. Infrared radiation has a longer wavelength than visible light and can be divided into FIR (5.6–1000 μm), middle-infrared radiation (1.5–5.6 μm), and near-infrared radiation (0.8–1.5 μm) [9]. Recent studies have shown that FIR therapy plays a beneficial role in the cardiovascular system [11]. FIR radiation improves endothelial function in patients with heart disease and increases access flow and patency of arteriovenous fistulas in hemodialysis patients [12,13,14]. Besides, FIR treatment can promote angiogenesis and microvascular blood flow in various animal models [11,15,16]. 

The present study aimed to test the hypothesis that FIR is effective in promoting diabetic wound healing. Therefore, this work aims to investigate the biological effect of FIR irradiation and the molecular mechanism of FIR on the healing of chronic wounds using the STZ-induced type I diabetes rodent wound model [17,18,19].

## 2. Materials and Methods

### 2.1. Animal Model

Four-month-old male Wistar rats were purchased from National Experimental Animals Production Center (Taipei, Taiwan). All experimental procedures and protocols involving animals were approved by the Institutional Animal Care and Use Committee on the protection of animals used for scientific purposes (IACUCA animal use protocol approval number: 107072) and in compliance with the Guide for the Care and Use of Laboratory Animals published by the National Institute of Health. Rats were housed in a 12-h light/dark cycle facility with a controlled temperature and kept with free access to water and food. A single dose of streptozotocin (STZ, 65 mg/kg, intraperitoneal injection, i.p.) in 0.1 M sodium citrate buffer was used in rats weighing 400 to 425 g, to generate a type I diabetic model according to previous reports [5,18,20]. The blood glucose levels from the tails were evaluated 1 and 2 weeks using a glucometer following STZ injection. Rats with glycemia more than 300 mg/dL were considered diabetic developed and then included for the following experiments. At two weeks after STZ induction, the rats were anesthetized with sodium pentobarbital (50 mg/kg, i.p.) and then subjected to full-thickness wounds. The skin flap tissue of the dorsum of the Wistar rats was excised to create a full-thickness skin defect with an area of 6 × 5 cm^2^ wound defect, which has been described previously [20]. Then use transparent Tegaderm (3M HealthCare, Borken, Germany) to briefly cover the wound until the start of FIR treatment. After the operation, the rat was returned to the cage in the animal containment room after regaining consciousness. The rodents were reared separately after operation.

### 2.2. Experimental Design

Male Wistar rats were divided into four groups (eight rats per group). Anesthesia was administered via inhalational general anesthesia of isoflurane along with an intramuscular injection of atropine (0.1 mg/kg) to reduce the secretion of saliva during and after the surgery. The rats were randomized into four treatment groups: (1) Sham, non-diabetic control; (2) DM control, diabetic control without treatment; (3) FIR-20, diabetic rats received 20 min FIR (the rats were wounded as described and then exposed to lamps for 20 min per session, triplicate in a week for 4 weeks) and (4) FIR-40, diabetic rats received 40 min FIR (the rats were wounded as described and then exposed to lamps for 40 min per session, triplicate in a week for 4 weeks). The rats were exposed to radiation from a WSTM TY301 FIR emitter (Far IR Medical Technology, Taipei, Taiwan) with an effective energy intensity of 0.13 mW/cm^2^. The wavelength of the light generated from the electrified ceramic plates was from 5 to 12 μm with a peak at 8.2 μm [1,10]. The radiator was set at a height of 20 cm above the cages for the indicated times (20 or 40 min). 

### 2.3. Monitoring of Blood Perfusion in Wound Area Using Laser Doppler

The blood perfusion in the wound area was measured using a peripheral microvascular laser Doppler flow-perfusion imager (Lisca-PIMII, Linköping, Sweden). The blood perfusion in the wound area was detected on days 4, 8, and 15 during the first two sessions of FIR. Laser Doppler blood flow measurement is performed using a camera-like device, which is used for two-dimensional mapping (imaging mode) and ceaseless recording (perfusion screen mode) of shallow tissue blood perfusion. In the imaging mode, a low-power (1-mW) laser beam with a wavelength of 670 nm effectively scanned the tissue at thousands of measurement points gradually through the optical fiber. In tissues, according to the well-known Doppler principle, light is scattered and changes frequency to interact with moving blood cells. The sample depth is two or three hundred micrometers. A small part of the back-dispersed and Doppler-widened light was identified by the light detector in the scanning head. For every estimation point, the Doppler expanding and size of the Doppler signal were determined and a sign was created that scales directly with tissue perfusion characterized as the result of red blood cell velocity and concentration. The outcomes are introduced as a two-dimensional color image on a PC monitor. The signal cannot be calibrated as the absolute value of blood flow; therefore, the output signal is expressed in arbitrary units.

### 2.4. Wound Healing Observation

On the 0, 14th, 28th, and 42nd days after treatment, the changes in wounds and granulation tissue growth were observed with naked eyes. The wound healing area was assessed once a week after the operation using the template technique, which has been described previously [20]. Meanwhile, all wounds were photographed with a digital camera (COOLPIX B700, Nikon, Tokyo, Japan). By macroscopic observation, a distinct demarcation line in the unhealed tissue area was traced onto the transparent graph paper. The traced area was cut and measured its weight, and calculated by the formula (1 − A1/A0) × 100%, where A0 is the original wound area (6 × 5 cm^2^) and A1 is the unhealed area. The area was calculated once a week until the whole wound had healed [21].

### 2.5. Histological Examination 

Histopathological observation of wound tissue structure on wound surface in the wound healing process was performed by HE staining experiment. Full-thickness 3-mm biopsies were obtained from the wound margin to investigate the pathological changes occurring in the wound at 1.5 weeks and 2.5 weeks post-treatment. Biopsy specimens were fixed in 10% formalin (Sigma-Aldrich, St. Louis, MO, USA) and embedded in paraffin. Sections for each group were stained with hematoxylin and eosin (H&E; Sigma-Aldrich). IHC semiquantitative staining was performed using horseradish peroxidase–diaminobenzidine (HRP–DAB) staining kit (R&D, Inc., Minneapolis, MN, USA) as described previously [18,22]. Polyclonal antibodies against CD45, VEGF, eNOS, EGF, 8-hydroxy-2-deoxyguanosine (8-OHdG), what’s more Ki-67 (Santa Cruz, Santa Cruz, CA, USA) were utilized as the primary antibodies, and the slides were brooded with these antibodies at 1:100 dilutions in PBS for 60 minutes. The sections were then brooded with biotinylated goat anti-rabbit antibody for 30 minutes. The specific combination of the secondary antibody and the primary antibody uses HRP to enzymatically convert the chromogenic substrate 3,3′-diaminobenzidine (DAB) into a brown precipitate for visualization. A Zeiss fluorescence microscope (Carl Zeiss, Gottingen, Germany) was used to mount, remove, cover glass and inspect the slices. 

### 2.6. Examination of Histomorphometric Markers

A Zeiss Axioskop 2 plus microscope (Carl Zeiss) was used to image tissue sections to evaluate the immunohistochemically stained cells. All pictures of every example were taken with a Cool CCD camera (SNAP-Pro cf. Computerized unit; Media Cybernetics, Silver Spring, MD, USA). As described previously, use Image-Pro Plus image analysis software (Media Cybernetics) to analyze the images. Acquire four random images from each selected area at 400× magnification. The quantity of immunopositive cells and the percentage of positively labeled cells to the total cells are shown. 

### 2.7. Data Management and Statistical Analysis

The experimental results are presented as the mean ± standard deviation (SD). Significant differences between experimental groups with replicates in each of the three independent experiments were analyzed by using the t-test, and *p* < 0.05 was considered statistically significant. 

## 3. Results

### 3.1. FIR in Enhancing Angiogenesis by Increase of Per-Wounding Blood Perfusion

**Figure 1:**
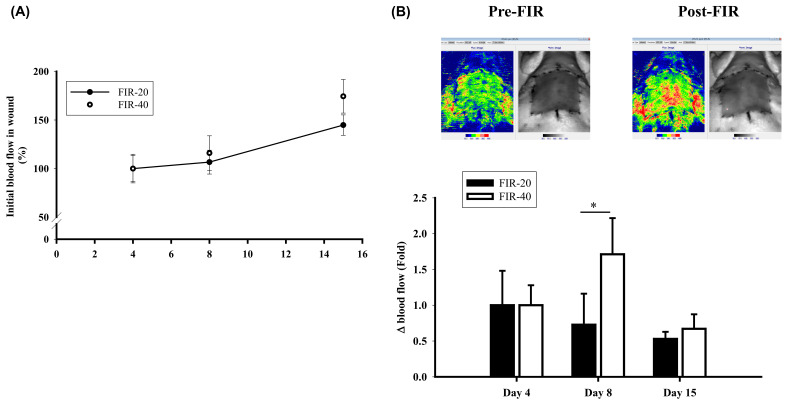
Far-infrared radiation (FIR) treatment in enhancing blood perfusion in the diabetic wound area. The blood perfusion in the wound area was detected by laser Doppler before (pre-FIR) and 1 h after the FIR (post-FIR) at the early and the late stage of treatment. (**A**) The initial blood flow in wounds was 365.38 ± 132.14 PU/wound for FIR 20 and 308.25 ± 82.99 PU/wound for FIR 40 on Day 4, which was set as 100%. (**B**) Upper: Laser Doppler Imager before (Pre-FIR) and after FIR treatment (Post-FIR). Lower: The change of blood flow was calculated by the expression (PostFIR − PreFIR)/PreFIR × 100% after FIR 20 and 40 min treatment respectively. The FIR-40 group showed a significant increase in wound area perfusion at the late phase after the FIR, * *p* < 0.05.

A previous study showed that FIR, independent of its thermal effects, enhances blood perfusion indicating that FIR irradiation could regulate angiogenesis. After far-infrared treatment, laser Doppler was used to detect blood perfusion in the wound area. The Doppler signal was produced as the velocity of red blood cells expressed in arbitrary units and is related to the tissue per-fusion in the wound area. The initial blood flow of FIR 40 groups is 9.29% more than FIR 20 on D8; 29.6% more than FIR 20 on Day 15. On Day 8, blood flow increased 70% after 40 min FIR treatment. The blood perfusion in the wound area revealed no significant difference between FIR-20 and FIR-40 groups on day 4 after FIR. However, the FIR-40 FIR-treated group still showed a significant increase in wound area perfusion on Day 8 after the FIR compared to FIR-20 FIR-treated diabetic rats (Figure 1B). These results indicated that FIR increases topical blood perfusion and facilitates the process of chronic wound healing.

### 3.2. FIR Enhanced Diabetic Wound Healing

**Figure 2:**
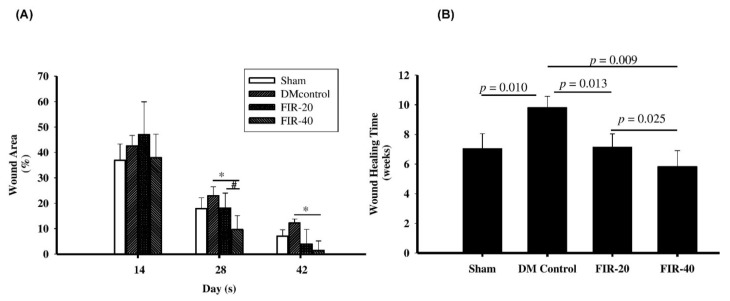
Far-infrared radiation (FIR) in accelerating wound healing in diabetic rats. (**A**) Wound size difference. Compare with DM, * *p* < 0.05; FIR-20 vs. FIR-40, # *p* < 0.05. (**B**) Average complete healing time. The complete wound healing time was significantly faster in the FIR group with 20 min (FIR-20) and 40 min (FIR-40) than the diabetic control group without treatment (*p* < 0.001). Nonetheless, the wound healing time in the FIR-20-treated group was still significantly longer than that in the FIR-40-treated rats with two sessions (*p* = 0.025).

The in vivo experimental results revealed that the wound size was significantly reduced during the wound healing process in the FIR-treated rats compared to the diabetic control rats. On the 0, 14th, 28th, and 42nd days after treatment, the changes in wounds and granulation tissue growth were observed and wound sizes were significantly reduced on the 28th and 42nd days post-treatment in the FIR-20 and FIR-40 groups compared with diabetic control (Figure 2A, *p* < 0.005). The complete wound healing time was significantly faster in the FIR-20 and FIR-40 groups than in the diabetic control group without treatment (*p* < 0.001) (Figure 2B). Nonetheless, the wound healing time in the FIR-20-treated group was still significantly longer than that in the FIR-40-treated rats (7.14 ± 0.90 weeks vs. 5.83 ± 1.08 weeks, *p* = 0.025). This result indicated that treatment with the optimal time length of FIR could promote faster diabetic wound healing.

### 3.3. FIR Suppressed the Inflammatory Response

**Figure 3:**
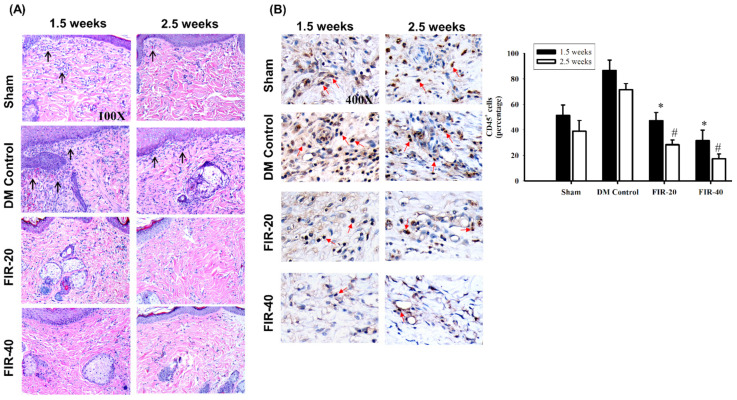
Far-infrared radiation (FIR) treatment suppressed the early inflammatory responses and reduced CD45+ cells in periwound edges. (**A**) The specimens harvested from the periwound area were histologically examined. H&E staining revealed that leukocyte infiltration (black arrows) from the dermis to the subcutaneous muscular layers was markedly reduced in the FIR-20-treated and FIR-40-treated diabetic rat groups at the early phase (1.5 weeks) of post-treatment compared to that in the diabetic controls and consistently decreased at the late phase (2.5 weeks) after treatment among the FIR-treated groups and the diabetic control group without treatments. (**B**) IHC staining indicated that CD45+ expression (red arrows) was significantly decreased in the FIR-20- and FIR-40-treated groups at the early (1.5 weeks) and late-phase (2.5 weeks) after treatment among the FIR-treated groups and the DM control group without treatments. Summary of CD45+ IHC staining in periwound edges after FIR-treatment. Compare with DM, * *p* < 0.05 (at 1.5 weeks); # *p* < 0.05 (at 2.5 weeks). Magnification, 100×.

The biopsy specimens retrieved from the wound edge area were histologically examined after the wounding. Hematoxylin and eosin staining revealed that leukocyte infiltration from the dermis to the subcutaneous muscular layers was markedly reduced in the FIR-treated diabetic rat groups at 1.5 weeks and 2.5 weeks post-treatment compared to that in the diabetic controls (Figure 3A). The IHC staining indicated that CD45+ expression was significantly decreased in the FIR-20- and FIR-40-treated diabetic rats groups when compared with rats in the DM control group (Figure 3B). This indicated diabetic rats with FIR treatment could regulate the adequate inflammatory response. 

### 3.4. FIR Suppressed Oxidative Damage

**Figure 4:**
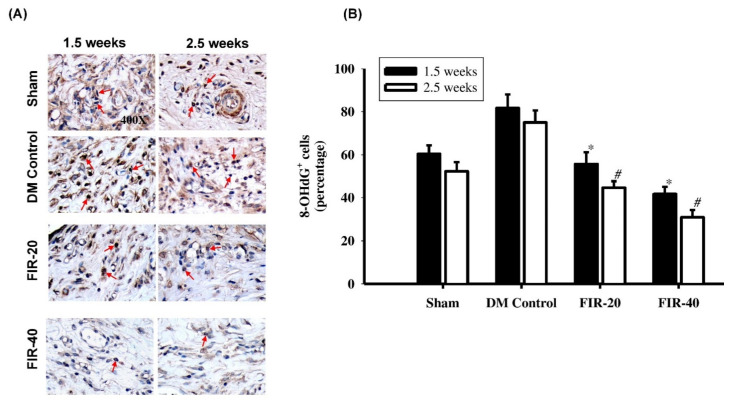
Far-infrared radiation (FIR) treatment for suppression of oxidative stress. (**A**) The oxidative damage indicated by the 8-OHdG level (red arrows) revealed a significant decrease in the diabetic wound healing process in the FIR-treated groups at the early (1.5 weeks) and the late (2.5 weeks) phase post-treatment compared to the DM control group. (**B**) Summary of 8-OHdG level in periwound edges after FIR-treatment. Furthermore, the rats in the FIR-40 group exhibited a marked decrease in 8-OHdG expression compared with the rats in the FIR-20 group. * *p* < 0.05 (at 1.5 weeks); # *p* < 0.05 (at 2.5 weeks). Magnification, 400×.

The oxidative damage indicated by the levels of 8-OHdG revealed a significant decrease in the diabetic wound-healing process in the FIR-treated diabetic groups at 1.5 weeks and 2.5 weeks post-treatment compared to the diabetic control group (Figure 4). Furthermore, the rats in the FIR-40 group exhibited a marked decrease in 8-OHdG expression compared with the rats in the FIR-20 group. 

### 3.5. FIR in Enhancing Cellular Proliferation and Regeneration

**Figure 5:**
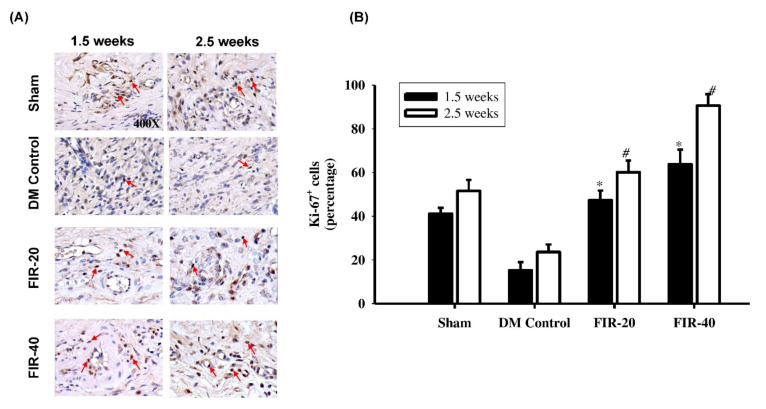
Far-infrared radiation (FIR) treatment in facilitating cell proliferation. (**A**) Analyze cell expansion based on the Ki-67 expression level (red arrow) at the wound edge, which is determined by performing HRP-DAB IHC staining. Expansion increment in Ki-67 expression, particularly in fibroblasts in the basal epidermal and subcutaneous layers, in the FIR-20- and FIR-40-treated diabetic rat groups at the early (1.5 weeks) and the late (2.5 weeks) stage after treatment compared with the DM control group (*p* < 0.001). (**B**) Summary of Ki-67+ cells in periwound edges after FIR-treatment. Furthermore, the rats in the FIR-40 group exhibited a marked increase in Ki-67 expression in the wound edge compared to that in the FIR-20 group. * *p* < 0.05 (at 1.5 weeks); # *p* < 0.05 (at 2.5 weeks). Magnification, 400×.

Cell expansion was examined based on the Ki-67 expression level at the wound edge, which was determined by performing HRP-DAB IHC staining. The staining results uncovered a critical expansion in Ki-67 articulation (Figure 5), particularly in the fibroblasts in the basal epidermal and subcutaneous layers, in the FIR-20 and FIR-40, FIR-treated diabetic rats at 1.5 weeks and 2.5 weeks post-treatment compared with the rats in the diabetic control group. Furthermore, the rats in the FIR-40 FIR-treated diabetic group exhibited a marked increase in Ki-67 expression in the wound edge compared to that in the FIR-20 FIR-treated group. This finding indicated that the long time length of FIR could increase cellular proliferation earlier and promote wound healing processing. 

### 3.6. FIR in Enhancing Angiogenesis by Upregulation of eNOS, VEGF and EGF Expression

**Figure 6:**
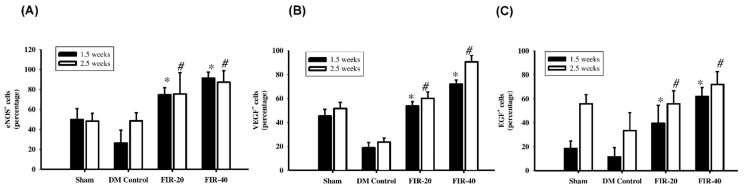
Far-infrared radiation (FIR) treatment in facilitating angiogenesis. The angiogenesis effect detected by eNOS (**A**), VEGF (**B**), and EGF (**C**) expression in the peri-wound tissue was investigated by using IHC staining. Expression of eNOS, VEGF, and EGF levels were increased, particularly in fibroblasts and endothelial cells, in the FIR-20- and FIR-40-treated diabetic groups at the early (day 10) and the late (day 17) stage post-treatment compared to the diabetic control group. In addition, eNOS, VEGF, and EGF expression were significantly increased in the FIR-40 group at the early and the late stage after treatment compared with the FIR-20 group. * *p* < 0.05 (at 1.5 weeks); # *p* < 0.05 (at 2.5 weeks).

During wound healing, angiogenic capillary sprouts invade the fibrin/fibronectin-rich wound clot and within a few days organize into a microvascular network throughout the granulation tissue. The angiogenic effect detected by eNOS, VEGF, and EGF expression in the periwounded tissue was investigated by using IHC staining. The eNOS, VEGF, and EGF levels were increased, particularly in fibroblasts and endothelial cells, in the FIR-20 and FIR-40 FIR-treated diabetic groups at 1.5 weeks and 2.5 weeks post-treatment compared to the diabetic control group (Figure 6). In addition, the eNOS, VEGF, and EGF expression levels along the wound edge were significantly increased in the FIR-40-treated diabetic rats at 1.5 weeks and 2.5 weeks compared with the FIR-20-treated diabetic group. These results indicated that the long time length of the FIR treatment group showed an enhanced angiogenic effect compared to the diabetic control group. 

## 4. Discussion

Unhealed chronic wounds are characterized by a prolonged inflammatory phase, delayed cellular proliferation, poor re-epithelialization, and impaired angiogenesis [3,23]. Since wound healing is impaired in diabetic patients, previous studies have reported various modalities, however, controversial results concerning wound healing in diabetic patients were be found. Therefore, researchers have attempted to design effective interventions that could accelerate the wound healing process. More and more physical therapies are used for enhancing the process of wound healing, such as extracorporeal shock-wave therapy, negative pressure wound therapy, etc. [5,18]. Studies have revealed that FIR therapy improves circulation, vessel endothelial function, and reduces atherosclerosis among diabetic patients undergoing hemodialysis [14]. 

The present study indicates that FIR therapy at least has two biological effects on chronic wound healing, including reduced inflammation and oxidative damage as well as promotion of proliferation and angiogenesis. Studies have shown that leukocyte-mediated inflammation is an important factor during the wound-healing process [20,24,25]. Histological analysis of the wound margin showed that the infiltration of inflammatory cells was attenuated on day 10 after the low strength and high strength FIR-treated rats compared with the diabetic controls. A previous study had shown that oxygen radicals were significantly increased in diabetic rats [17]. The expression of 8-OHdG was also markedly increased in the diabetic endothelium and subintima compared to that in normal vessels [17]. To assess the oxidative change of chronic wounds, we performed IHC staining of 8-OHdG. The results revealed that the level of 8-OHdG was significantly decreased in the low strength and high strength FIR-treated groups during the diabetic wound healing process. Furthermore, the rats in the high strength FIR-treated group exhibited a marked decrease in 8-OHdG expression compared to that in the low strength FIR-treated diabetic rats. These results indicated that high strength FIR treatment more effectively and significantly suppressed 8-OHdG expression than low strength. This may be considered a further validation of reduced inflammatory responses in FIR-treated rats compared with the diabetic controls. Further studies should be needed to provide more semi-quantification data analysis, such as TNFα or IL-2 expressions, to elucidate the inflammatory-related pathways in FIR facilitating wound healing.

Cellular proliferation and regeneration were examined in terms of Ki-67 expression in the wound margin [4,20]. The cell proliferation marker Ki 67 is expressed by proliferating cells in the late G1, S, and G2/M phases of the cell cycle. The nuclear localization of Ki 67 and its specific association with the cell cycle shows its significance in the regulation of cell division during the wound proliferation phase. The evidence from this study implied that whether FIR-20 or FIR-40 FIR-treated groups had a marked increase in Ki-67 expression, particularly in the fibroblasts and basal epidermal layers. Furthermore, the rats with FIR-40-treated diabetic rats exhibited a marked increase in Ki-67 expression in the wound margin compared to the rats in the FIR-20-treated group. This finding indicated that the long time length of the FIR treatment was more effective in increasing cellular proliferation and facilitating the process of chronic wound healing.

Angiogenic factors play an important role in the wound healing process. ENOS is an activated enzyme required for angiogenesis, and VEGF is recognized as the most important biological indicator of angiogenesis; EGF is also considered to be an important growth factor for important vascular endothelial cells in addition to being related to angiogenesis. In the present study, expression of eNOS, VEGF, and EGF in the wound edge after treatment was investigated by using IHC staining. The experimental results indicated that expression of angiogenic factors, eNOS, VEGF, and EGF was significantly elevated in the wound margin area, particularly in endothelial cells and fibroblasts, at 1.5 weeks and 2.5 weeks after the low strength or high strength FIR treatment compared with the diabetic control group. Moreover, the eNOS, VEGF, and EGF expression levels were increased in the FIR-40-treated group compared with the FIR-20 treatment group. This result demonstrated that the long time length of the FIR treatment was more effective in enhancing angiogenesis and the induction of neovascularization in the transitional zone of the wound margin. Additionally, blood circulation detected by a laser Doppler flow-perfusion imager showed no significant differences existed in early tissue perfusion in FIR-20- or FIR-40-treated groups. However, a significant increase existed in late tissue perfusion between the FIR-20-treated group and the FIR-40-treated diabetic rats. This finding indicates that FIR-40 has late enhanced topical blood perfusion and facilitates the wound healing process. This demonstrates that FIR could significantly increase blood perfusion and epithelialization during wound healing in a model of diabetes.

However, there are still some limitations to this study. This study is limited by the small number of animals, thereby resulting in a relatively low statistical power. Additionally, this is a very early-stage study concerning the comparison of low strength and high strength FIR for wound healing in a rodent model of diabetes. Many additional experiments are required to overcome the limitations of our experimental design to elucidate the mechanical effects such as the inflammatory activity of systemic effects detected by flow cytometry, or using ELISA, as well as more wound healing-related molecules of per-wounding tissue expressions such as HIF-1, angiogenesis-related pathway [25].

In summary, this study demonstrated that both time lengths of FIR enhanced diabetic wound healing by their effects on wound epithelium, suppression of the inflammatory response, facilitation of cellular proliferation, and effects on angiogenesis and oxidative damage. However, the optimal time length of the FIR treatment was more effective in the acceleration of wound healing than the diabetic control. This technique represents a feasible method for accelerating wound healing or augmenting compromised tissue circulation and diabetic ulcers.

## 5. Conclusions

**Figure 7:**
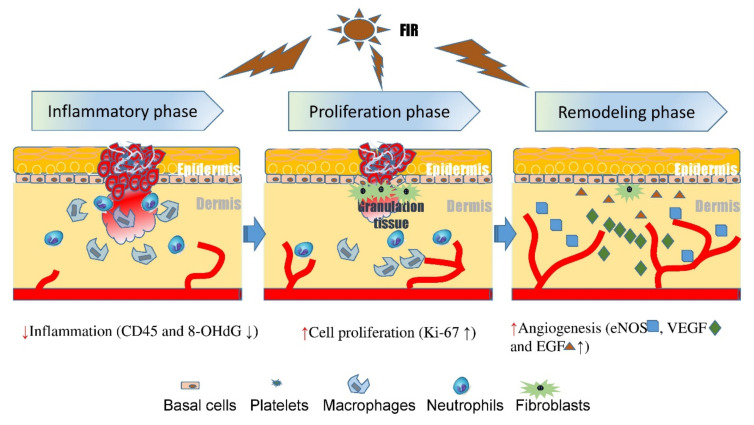
Proposed the biomechanisms of far-infrared radiation (FIR) treatment suppress the leukocyte infiltration, decreasing inflammatory response (e.g., CD45+, 8-OHdG), enhancing cellular proliferation (e.g., Ki-67+ cells) and angiogenic factors (e.g., eNOS, VEGF, EGF), resulting in inducing epithelialization and wound healing.

Our results have revealed that both time lengths of FIR enhanced diabetic wound healing by their effects on wound epithelium, suppression of the inflammatory response, facilitation of cellular proliferation, and effects on angiogenesis and oxidative damage (Figure 7). However, the optimal time length of the FIR treatment was more effective in the acceleration of wound healing than the diabetic control. This technique represents a feasible method for accelerating wound healing or augmenting compromised tissue circulation and diabetic ulcers.

## Data Availability

Not applicable.
